# The Influence of Magnetic Fields on the Electrical Conductivity of Membranes based on Cotton Fabric, Honey, and Microparticles of Carbonyl Iron and Silver

**DOI:** 10.3390/ma16051995

**Published:** 2023-02-28

**Authors:** Ioan Bica, Gabriela-Eugenia Iacobescu

**Affiliations:** 1 Advanced Environmental Research Institute, West University of Timisoara, Bd. V. Parvan, Nr. 4, 300223 Timisoara, Romania; 2 Department of Physics, University of Craiova, Str. A. I. Cuza, Nr. 13, 200585 Craiova, Romania

**Keywords:** magnetically active membranes, magnetic dipole, carbonyl iron, silver microparticles, electrical conduction

## Abstract

In the present work, we report that the manufacturing of new environmentally friendly and low-cost materials with electrical conductivity can be roughly and finely tuned by an external magnetic field for technical and biomedical applications. With this aim in mind, we prepared three types of membranes based on cotton fabric impregnated with bee honey, carbonyl iron microparticles (CI), and silver microparticles (SmP). In order to study the influence of the metal particles and the magnetic field on the electrical conductivity of membranes, electrical devices were made. Using the “volt-amperometric” method, it was found that the electrical conductivity of the membranes is influenced by the mass ratio ($m_{\mathrm{CI}}$:$\;m_{\mathrm{SmP}}$) and by the B values of the magnetic flux density. It was observed that in the absence of an external magnetic field, adding microparticles of carbonyl iron mixed with silver microparticles in mass ratios ($m_{\mathrm{CI}}$:$\;m_{\mathrm{SmP}}$) of 1:0, 1:0.5, and 1:1 causes the electrical conductivity of the membranes based on cotton fabrics impregnated with honey to increase 2.05, 4.62, and 7.52 times, respectively, compared with that of the membrane based on cotton fabrics impregnated with honey alone. When applying a magnetic field, the electrical conductivity of the membranes with microparticles of carbonyl iron and silver increases with increasing magnetic flux density *B*. We conclude that the membranes are very good candidates for the fabrication of devices to be used in biomedical applications due to the possibility of remote, magnetically induced release of the bioactive compounds from honey and silver microparticles into the area of interest during medical treatment.

## 1. Introduction

Membranes (Ms) are a field of research closely connected with addressing the needs of society, such as the treatment of water and wastewater from agriculture [1,2], the isolation and purification of organic products [3,4], in hemodialysis [5,6], the isolation and purification of components from fluid mixtures [7,8], as well as membranes with magnetodielectric properties for technical and biomedical purposes [6,9,10]. It is known that due to flavonoids [11,12], bee honey has antitumor, antioxidant, anti-inflammatory, and immunomodulatory effects. Through its analgesic, anti-inflammatory, anticarcinogenic, and antibacterial activity, honey has been shown to have a major impact on patients’ quality of life and nutritional status by promoting tissue epithelization and healing of chemoradiotherapy-induced lesions. Superior to many other natural agents, bee honey can be used successfully in both the prevention and treatment of oral mucositis. Currently [13,14], the use of bee honey is supported and recommended in the management of oncological toxicity.

On the other hand, all living beings are subject to the permanent action of the terrestrial magnetic field. Moreover, relatively recent studies have reported the therapeutic effects of magnetic fields and shown how it can be used in medical therapies [15,16].

Silver nanoparticles, due to their antimicrobial effect, antifungal action, and antiviral activity [17,18], are widely used in medicine. Nevertheless, silver micronanoparticles exert their antimicrobial effect by damaging microbial membranes through their physicochemical attachment to the cell surface, leading to subsequent structural and functional changes, including gap formation, membrane destabilization, membrane perforation, and cytoplasmic leakage [19,20]. Moreover, silver particles are capable of damaging the subcellular structure, leading to the release of free Ag+ ions and the subsequent generation of reactive oxygen species, resulting in the inactivation of proteins, enzymes, and nucleotides [19]. Recent studies, such as [21], point out that carbonyl iron tablets are much better tolerated by the human body than iron sulfate tablets in the treatment of anemia, participating in the replenishment of the iron ion reservoir of the human body and avoiding the generation of mental illnesses. Based on these considerations, the manufacturing of Ms in which the therapeutic effects of bee honey, carbonyl iron mixed with silver microparticles, and the magnetic field are working together is a real challenge. 

Recently, the manufacture of a magnetodielectric material based on cotton microfibers with bee honey, carbonyl iron microparticles, and silver microparticles was reported in [22]. Electrical devices (EDs) were made from these composites. EDs were placed in a medium-frequency electric field (10–30 kHz) superimposed on a static magnetic field with magnetic flux density values of 0 mT, 200 mT, and 400 mT. It was observed that in the presence of the magnetic field, the relative dielectric permittivity (through the equivalent electrical capacity of the electrical devices) and the electrical conductivity (through the equivalent electrical resistance) are sensitively influenced by the magnetic field. We consider that, on the one hand, for practical applications, the use of the medium-frequency electric field increases the cost price of electrical devices, while on the other hand, avoiding interference with alternative electric fields is difficult to achieve. The simplification of the electrical devices made with this type of membrane requires the removal of the medium frequency generator. For these reasons, in the present work, three types of membranes based on cotton fabrics were prepared via impregnation with either bee honey alone ($M_{0}$), bee honey and carbonyl iron microparticles ($M_{1}$), or bee honey mixed with carbonyl iron and two different masses of silver microparticles ($M_{2}$,$\;M_{3}$). The mass of silver microparticles in $M_{3}$ is twice that of $M_{2}$. For a constant voltage applied to electrical devices made with these membranes, it is shown that the electrical conductivity can be roughly adjusted by using metal microparticles. For membranes containing microparticles of carbonyl iron, the electrical conductivity can be fine-tuned using the magnetic field. Consequently, the thermal transport of the honey components and the iron and silver ions in thermal treatments can be achieved using such low-cost electrical devices.

## 2. Materials and Methods

### 2.1. Materials for Ms Manufacture

**Figure 1:**
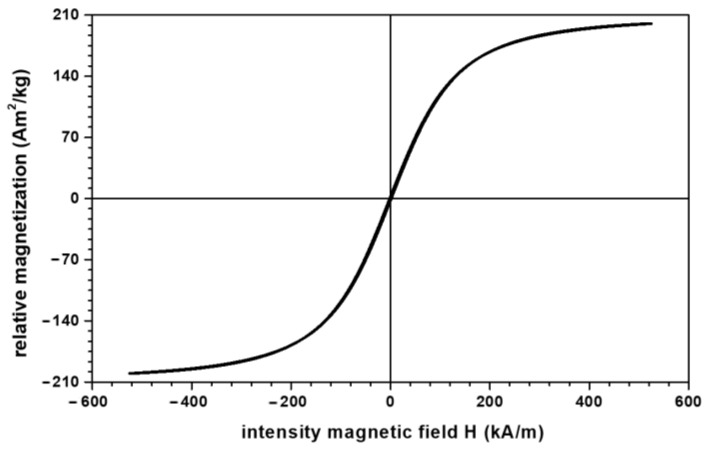
Magnetization slope of CI microparticles.

The materials needed for Ms manufacture were as follows:

(a) Sterile compresses (GBs) were produced by Shaoxing Gangfeng Hospital Products Co Ltd. (Gaobu Town, Shaoxing, Zhejiang, China) and purchased through Labomed Pharma SA (Bucuresti, Romania). The GBs were made using cotton microfibers (CmF) with 100% purity and mass density of $\rho_{\mathrm{CmF}}=0.735\;g/{\mathrm{cm}}^{3}$ at a temperature of 22 °C and a relative air humidity of 60%.
(b) Bee honey (HB) was purchased through Biosimttera Cluj (Cluj-Napoca, Romania) with mass density $\rho_{\mathrm{HB}}=1.496\;g/{\mathrm{cm}}^{3}$ at a temperature of 22 °C.
(c) Microparticles of carbonyl iron (CI) from Sigma-Aldrich Chemie GmbH (Taufkirchen, Germany), product code C-3518, with a minimum purity of 99.7% and an average diameter $d_{\mathrm{CI}}\approx 5\;\mu m$ at a temperature of 24 °C. The electrical resistivity of CI microparticles is $\rho_{\mathrm{CI}}=9.71\;\mu \Omega \cdot \mathrm{cm}$ at a temperature of 20 °C, and the bulk density of CI microparticles is $\rho_{\mathrm{CI}}=3.512\;g/{\mathrm{cm}}^{3}$ at a temperature of 22 °C. From the magnetization slope of CI microparticles, recorded using the experimental set-up described in [23], we measured the relative saturation magnetization as $\sigma_{s\mathrm{CI}}=200\;A\cdot m^{2}/\mathrm{kg}$ at H≥500 kA/m (Figure 1).
(d) Silver microparticles (SmP), from Sigma-Aldrich (Saint Louis, MO, USA), product code 327085, with a minimum purity of 99.9%, average diameter $d_{\mathrm{SmP}}\approx 3\;\mu m,$ electrical resistivity of $59\times 10^{-6}\;\mu \Omega \cdot \mathrm{cm},$ and bulk density $\rho_{\mathrm{SmP}}=1.336\;g/{\mathrm{cm}}^{3}$ at a temperature of 20 °C.

### 2.2. Ms Manufacturing

For the manufacture of Ms from GBs, four packages containing four tissues were formed. Each package had the dimensions 3$0\times 30\times 0.40{\;\mathrm{mm}}^{3}$, mass $m_{\mathrm{GB}}=0.200\;g$ and volume of microfibers $V_{\mathrm{CmF}}=m_{\mathrm{GB}}/\rho_{\mathrm{CmF}}\approx 0.272{\;\mathrm{cm}}^{3}.\;$Quantities of HB, CI, and SmP were weighed to the values shown in Table 1. After being mixed and homogenized for 30 min, the magnetic solutions $S_{i}\;\left(i=0,1,2,3\right)$ were obtained, with the volume fractions as given in Table 1.

Here, $m_{\mathrm{HB}}$, $m_{\mathrm{CI}}$, $m_{\mathrm{SmP}}$, $\varphi_{\mathrm{HB}}$, $\varphi_{\mathrm{CI},}$ and $\varphi_{\mathrm{SmP}}$ are the masses and mass fractions of HB, CI microparticles, and SmP microparticles, respectively.

**Figure 2:**
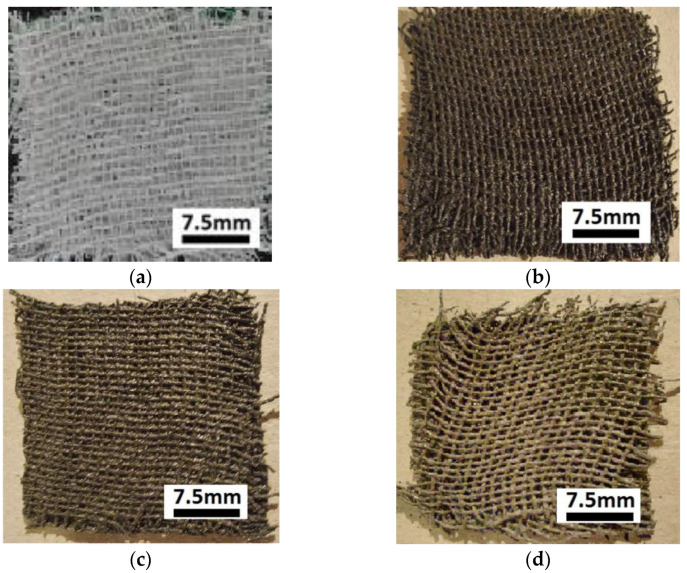
Images of $M_{i}\;\left(i=0,1,2,3\right)$ membranes: (**a**) $M_{0}$; (**b**) $M_{1}$; (**c**) $M_{2}$; (**d**) $M_{3}$.

Next, the GBs packages were immersed as follows: the first package in the $S_{0}$ suspension, the second package in the $S_{1}$ suspension, the third package in the $S_{2}$ suspension, and the fourth package in the $S_{3}$ suspension. At the end of this stage, the membranes $M_{i}\;\left(i=0,1,2,3\right)\;$shown in Figure 2 were obtained.

Each membrane was weighed on an analytical balance (ALN 60, produced by AXIS, Gdansk, Poland), and the obtained masses $m_{M_{i}}\;\left(i=0,1,2,3\right)$ are shown in Table 1. Given $m_{\mathrm{GB}}=0.200\;g$ and the values of $\varphi_{\mathrm{HB}}$, $\varphi_{\mathrm{CI}}$, and $\varphi_{\mathrm{SmP}}$ from Table 1, we calculated $m_{M_{i}}-m_{\mathrm{GB}}\;\left(i=0,1,2,3\right)$ to obtain the values of $m_{\mathrm{HB}}$, $m_{\mathrm{CI}}$, and $m_{\mathrm{SmP}}$ given in Table 2.

From $m_{\mathrm{HB}}$, $m_{\mathrm{CI}}$, and $m_{\mathrm{SmP},}\;$and using the abovementioned values of$\;\rho_{\mathrm{HB}}$, $\rho_{\mathrm{CI}}$, and $\rho_{\mathrm{SmP}}$, we calculated the volumes$\;V_{f},$ $V_{\mathrm{HB}}$, $V_{\mathrm{CI}}$, and $V_{\mathrm{SmP}}$ (Table 3). If we divide the volumes$\;V_{f},$ $V_{\mathrm{HB}}$, $V_{\mathrm{CI}}$, and $V_{\mathrm{SmP}}$ by the volumes $V_{M_{i}}\;\left(i=0,1,2,3\right)$ we obtain the volume fractions $\Phi_{f},$ $\Phi_{\mathrm{HB}}$, $\Phi_{\mathrm{CI}}$, and $\Phi_{\mathrm{SmP}}$ (Table 3).

**Figure 3:**
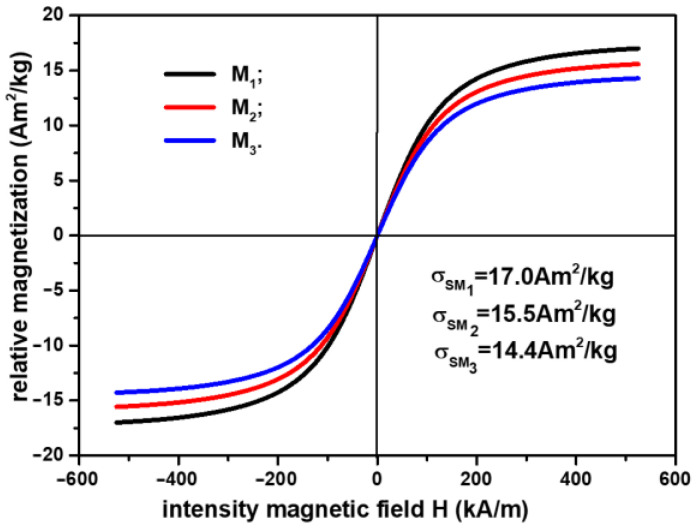
Magnetization slopes of membranes $M_{i}\;\left(i=1,2,3\right)$.

In order to plot the magnetization slopes of the membranes, $M_{i}$, we took into account the result reported in [24], namely that the relationship between the relative saturation magnetization of the CI microparticles, $\sigma_{s\mathrm{CI}}$, and the saturation magnetization of Ms, $\sigma_{sM_{i}}\;\left(i=1,2,3\right)$, can be represented by the equation $\mu_{0}\sigma_{sM_{i}}=\Phi_{{\mathrm{CI}}_{i}}\;\mu_{0}\sigma_{s\mathrm{CI}}\;\left(i=1,2,3\right)$, where $\mu_{0}$ is the magnetic permeability of the vacuum and $\Phi_{\mathrm{CI}}$ is the volume fraction of CI microparticles (from Table 3). With these considerations, the magnetization slopes of Ms are represented in Figure 3.

From Figure 3, it can be observed that the relative saturation magnetization $\sigma_{sM_{i}}\;\left(i=1,2,3\right)$ is strongly influenced by the component elements of the membranes $M_{i}\;\left(i=1,2,3\right)$. By increasing the amount of SmP microparticles, the weight of the magnetizable phase is decreased, as shown in Table 3. Thus, compared with $M_{1}$, where the relative saturation magnetization is $\sigma_{sM_{1}}=17.0{\;\mathrm{Am}}^{2}/\mathrm{kg},\;$the relative saturation magnetization decreased by about 9% for $M_{2}$ and about 16% for $M_{3}$.

The surface morphologies and elemental compositions of the obtained samples were highlighted using the electronic scanning microscope Inspect S (SEM) from FEI Europe B.V., Eindhoven, the Netherlands, equipped with an X-ray energy-dispersive spectrometer (EDX). All studied samples were analyzed in low vacuum mode using an LFD detector with a spot value of 3.5, pressure of 30 Pa, and high voltage of 30 kV.

**Figure 4:**
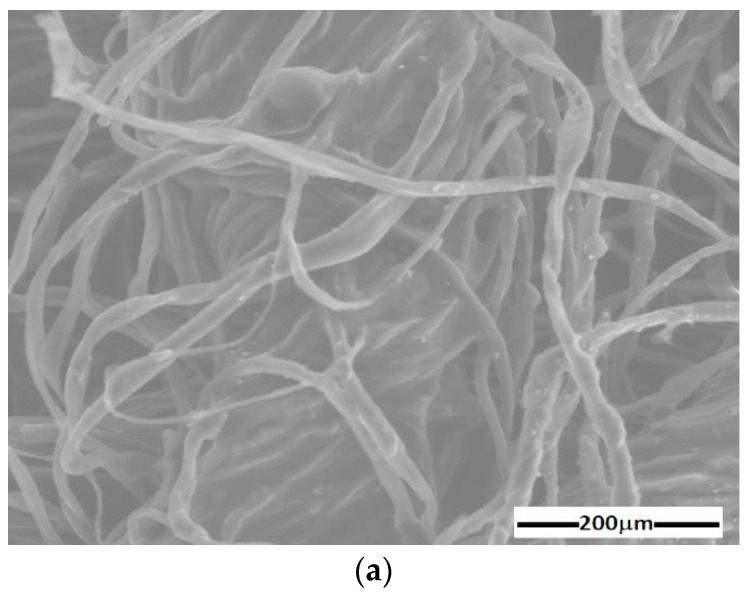

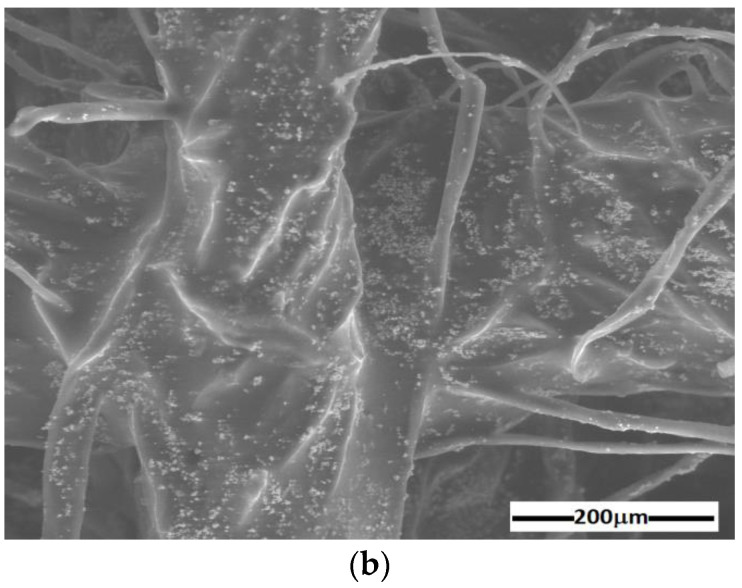
SEM image of cotton microfiber membranes with (**a**) s honey and (**b**) honey, CI, and silver microparticles.

**Figure 5:**
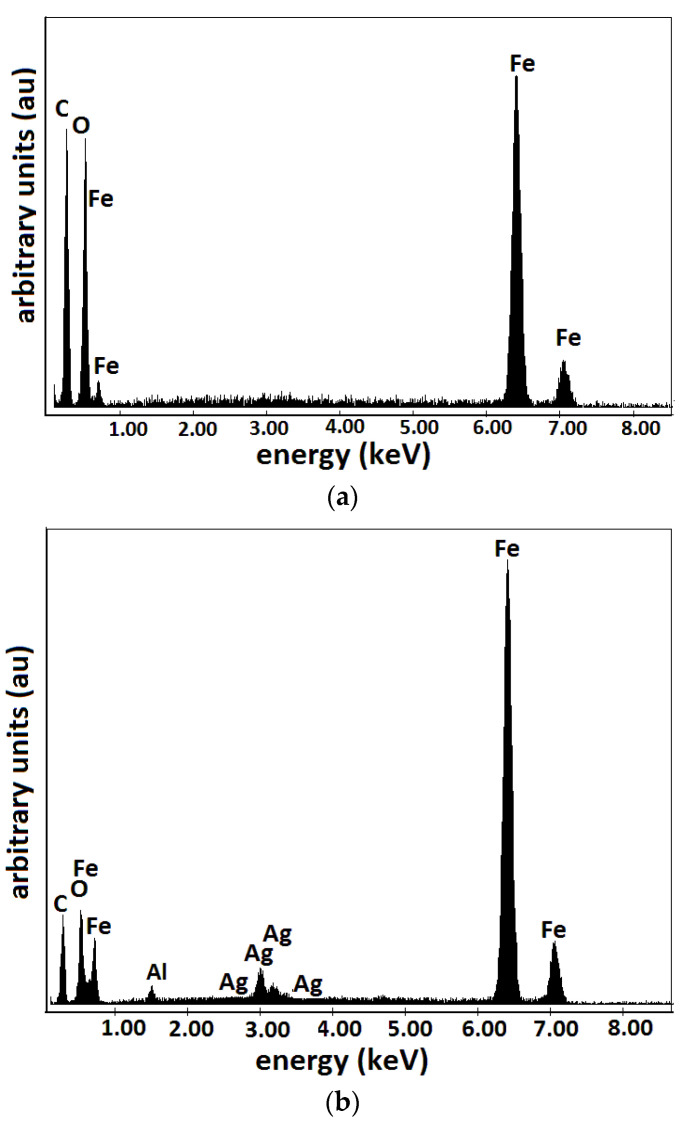
EDX elemental analysis of Ms microfibers containing: (**a**) CI; (**b**) CI and silver microparticles.

The results are represented in Figure 4 and Figure 5. The random distribution of microfibers in the base liquid (honey) can be observed in SEM images of the cotton fabric microfibers with honey (Figure 4a). From Figure 4b, it can be seen that the metal particles adhere to the microfibers and have entered the spaces between the cotton microfibers through capillarity action. From Figure 5, it can be seen that the membranes $M_{i}\;\left(i=0,\;1,\;2,\;3\right)$ are of high purity. Membrane $M_{0}$ contained C and O atoms. Similarly to $M_{0}$, membrane $M_{1}$ contains C and O atoms but also Fe atoms from the CI microparticles. It can be seen that membranes $M_{2}$ and $M_{3}$ (Figure 5b) contain C, O, and Fe atoms, similar to membrane $M_{1}$, with additional Ag atoms from SmP microparticles.

### 2.3. ED Manufacturing

The following materials were used to manufacture EDs:

- A textolite plate coated with copper foil (PCu) with dimensions of 100 × 75 × 0.8 mm^3^, from Electronic Light Tech (Romania). The plate (PCu) was based on epoxy resin, FR40type, reinforced with fiberglass, and copper foil of 35 μm thickness.
- $M_{i}\;\left(i\;=0,\;1,\;2,\;3\right)$ membranes (Table 3) with dimensions of 30 × 30 × 0.4 mm^3^.

**Figure 6:**
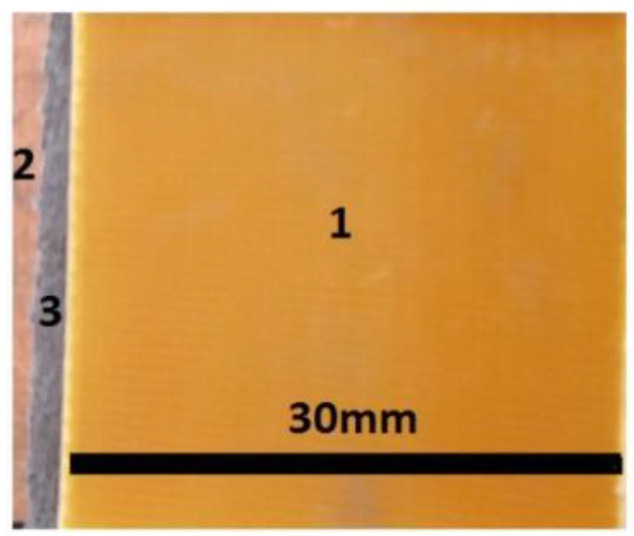
The components of electrical device: 1—the surface of the epoxy resin board reinforced with glass fibers; 2—copper foil; 3—membrane $M_{i}\;\left(i=0,1,2,3\right)$.

For the manufacture of four EDs, eight pieces with dimensions 30 × 30 × 0.80 mm^3^ were cut from a PCu board. Between the two plates, on the copper side, we placed $M_{i}\;\left(i=0,1,2,3\right)$ membranes. They attached as a result of the adhesive properties of HB. Repeating these steps for each membrane, the devices ${\mathrm{ED}}_{i}\;\left(i=0,1,2,3\right)$ were obtained, with the shape as shown in Figure 6.

### 2.4. Experimental Set-Up and Measurements

**Figure 7:**
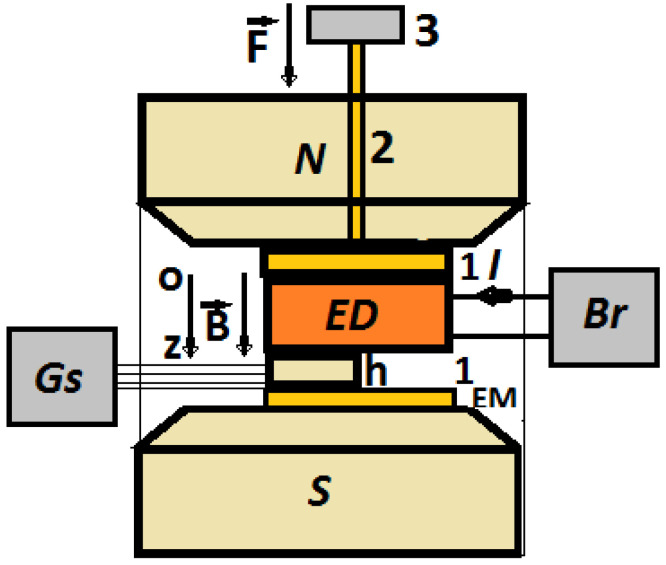
Experimental set-up (overall configuration): EM—electromagnet; N and S—magnetic poles; Br—impedance meter; Gs—gaussmeter; h—Hall probe; **B**—magnetic flux density vector; **F**—gravity force; ED—electrical device; 1—non-magnetic plate, 2—non-magnetic spindle, 3—non-magnetic weight.

The experimental set-up, as shown in Figure 7, consists of an electromagnet between the poles to which EDs are inserted one by one.

The fixing of the ED and the Hall probe was performed by mechanical tensioning. The mechanical tensioning of the ED device and the Hall probe was achieved through a non-magnetic mass (3 from Figure 7) acting on a non-magnetic plate (1 from Figure 5b) through the spindle (2) from Figure 7. Due to the gravity force (F=9 N) acting on the spindle, mechanical tension of $\tau =10\;\mathrm{kN}/m^{2}$ developed on the surface of the ED. The effect of this pressure was the result of good electrical contact between the surface of the membranes $M_{i}\;\left(i=0,1,2,3\right)$ and the electroconductive surfaces of the EDs (the copper electrodes).

The electromagnet was powered by a DCS source, type RXN-3020D, produced by ZHAOXIN (Shanghai, China). Between the N and S poles, the magnetic flux density could be continuously tuned by varying the intensity of the electric current, produced by the DCS source, through the electromagnet coil. The control of the magnetic flux density value was carried out by means of the Hall probe, h in Figure 7, connected to the gaussmeter, type DX-102, produced by DexingMagnet (Xiamen, China). The intensity of the electric current, I, through the EDs, was the result of fixed direct voltages, U, from the direct current source of the impedance meter Br, type E7-20, produced by MNIPI (Minsk, Belarus). The electric current intensity, I, was measured using a microammeter provided by Br. The measured quantities were recorded in a calculation unit (not shown in Figure 5b) and processed using appropriate software (Origin 9.0). 

## 3. Results and Discussion

The effects of the magnetic field on the electrical conductivity of the membranes $M_{i}\;\left(i=0,1,2,3\right)$ were studied using the experimental set-up in Figure 7.

For each electrical device, ED, fixed between the N and S poles of the electromagnet, EM, the intensity of the electric current, *I*, was measured at the voltage $U=2{\;V}_{\mathrm{dc}}$ generated by the internal source of Br.

The measurement of the electric current intensity, *I*, through ${\mathrm{ED}}_{i}\;\left(i=0,1,2,3\right)$, was performed in both the absence and presence of the magnetic field. 

### 3.1. The Study of the Electrical Conductivity of Ms in the Absence of the Magnetic Field

At the terminals of the EDs, the voltage $U=2{\;V}_{\mathrm{dc}}$ was fixed, and in the absence of the magnetic field the values, I, of the electric current intensity were measured. The measured values are shown in Table 4. 

The experimental data regarding the intensity of the electric current through the EDs in Table 4 suggest that the introduction of metal microparticles into the cotton fabric impregnated with bee honey influences the electrical conductivity of Ms. In order to quantitatively evaluate this influence, we can consider the EDs as linear resistors, and their electrical resistance $R_{0}{}_{i}\;\left(i=0,1,2,3\right)$ can thus be approximated by

(1) $$R_{0_{i}}=\frac{U}{I_{i}}=\frac{h_{0}}{\sigma_{0}{}_{i}\cdot L\cdot l}\;;\;\left(i=0,1,2,3\right),$$

where U is the electric voltage; $I_{i}\;\left(i=0,1,2,3\right)$ is the intensity of the electric current; $\sigma_{i}\;\left(i=0,1,2,3\right)$ is the electrical conductivity of the membrane $M_{i}\;\left(i\;=0,1,2,3\right)$ with the volume fraction of conductive microparticles given in Table 3; and $h_{0}$, L, and l are the thickness, length, and width of Ms, respectively. 

The electrical conductivity of the membranes $M_{i}\;\left(i\;=0,1,2,3\right)$ can be obtained from Equation (1) as

(2) $$\sigma_{0}{}_{i}=\frac{I_{i}h_{0}}{ULl}\;;\;\left(i=0,1,2,3\right).$$

If in Equation (2) we use $h_{0}=0.40\;\mathrm{mm}$, L=30 mm, l=25 mm, and $U=2V_{\mathrm{dc}}$ we find that:

(3) $$10^{6}\cdot \sigma_{0}{}_{i}\left(\Omega^{-1}\cdot m^{-1}\right)=\frac{4}{15}I_{i}\left({\mu A}_{\mathrm{dc}}\right);\;\left(i=0,1,2,3\right).$$

If we enter the $I_{i}$ values from Table 4 in Equation (3), we obtain the electrical conductivity values as shown in the fourth column of the same table. 

The results in Table 4 suggest that the insertion of metal microparticles into cotton fabric impregnated with bee honey led to an increase in electrical conductivity σ. With the electrical conductivity of $M_{0}$ chosen as a reference value, we observe from Table 4 that the electrical conductivity increased as follows:

- 2.19 ⁄ 0.64 ≈ 3.42 times for $M_{1}$;
- 4.93 ⁄ 0.64 ≈ 7.70 times for $M_{2}$;
- 8.03 ⁄ 0.64 ≈ 12.55 times for $M_{3}$.

The contribution brought by SmP microparticles to the increase in electrical conductivity was determined by taking the value of the electrical conductivity of membrane $M_{1}$ as a reference. Thus, with $\sigma_{0}{}_{1}=2.19\times 10^{-6}{\;\Omega }^{-1}\cdot m^{-1}$ as the reference value, we find from Table 4 that the electrical conductivity increased as follows:

- 4.93 ⁄ 2.19 ≈ 2.25 times for $M_{2}$;
- 8.03 ⁄ 2.19 ≈ 3.67 times for $M_{3}$.

### 3.2. Studying the Electrical Conductivity of Ms in the Presence of a Magnetic Field

**Figure 8:**
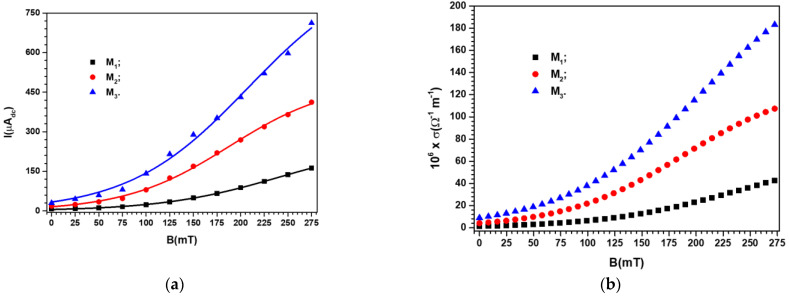
(**a**) The intensity, *I*, of the electric current through the devices ${\mathrm{ED}}_{i}\;\left(i=1,2,3\right)$, depending on the *B* values of the magnetic flux density (points—experimental data, lines—polynomial fits); (**b**) The electrical conductivity *σ* of the membranes $M_{i}\;\left(i=1,2,3\right)$ depending on the *B* values of the magnetic flux density.

Between the N and S poles of the electromagnet in Figure 7, the devices ${\mathrm{ED}}_{i}\;\left(i=0,1,2,3\right)$ were inserted one by one. The voltage value at the terminals of the devices ${\mathrm{ED}}_{i}\;\left(i=0,1,2,3\right)$ was maintained at the value $U=2{\;V}_{\mathrm{dc}}$. At Δ*t* = 60 s time intervals, after fixing the *B* values of the magnetic flux density, the values $I_{i}\;\left(i=0,1,2,3\right)$ of the electric current intensity were measured. As expected, the value of the intensity of the electric current through the device ${\mathrm{ED}}_{0}$ indicates that there is no influence from the magnetic field. Instead, the dependence of the electric current intensity on the *B* values of the magnetic flux density (increased in steps of 25 mT, up to a maximum of 275 mT) for the devices ${\mathrm{ED}}_{i}\;\left(i=1,2,3\right)$ are represented in Figure 8. 

It can be seen from Figure 8 that the electric current intensity values through the devices ${\mathrm{ED}}_{i}\;\left(i=1,2,3\right)$ were strongly influenced by the existence and concentration of metal microparticles in the body of the membranes $M_{i}\;\left(i=1,2,3\right)$. For the same membrane with metal microparticles, the electric current intensity values were sensitively influenced by the B values of the magnetic flux density. Thus, when the magnetic flux density increased from *B* = 25 to 275 mT, the increase in the value *I* of the electric current intensity was ~30 times for membrane $M_{1}$, ~25 times for membrane $M_{2,}$ and ~20 times for membrane $M_{3}$. In the case of membranes with mixtures of carbonyl iron and silver microparticles, the decrease in the rate of the *I* value of the electric current intensity is due to the decrease in the value of the volume fraction $\Phi_{\mathrm{CI}}$, which is ~10% in the $M_{2}$ membrane and ~18% in the $M_{3}$ membrane, as can be seen from Table 3.

For the description of the mechanisms that led to the results in Figure 8, we considered that in the devices ${\mathrm{ED}}_{i}\;\left(i=1,2,3\right)$, the CI microparticles are one-dimensional and uniformly distributed in the cotton fibers impregnated with bee honey. When the magnetic field is applied (moment *t* = 0 s), the CI microparticles transform, in a few moments, into magnetic dipoles. The magnetic dipoles form identical columns, equidistant and uniformly distributed in volume, in the direction of the magnetic field which is perpendicular to the surface of the membranes $M_{i}\;\left(i=1,2,3\right)$ [25]. Based on the model in [25], it is demonstrated that the magnetic force induced by the magnetic field in the volume of the membranes $M_{i}\;\left(i=1,2,3\right)$ can be calculated as

(4) $$F_{m_{i}}=-\frac{9\Phi_{{\mathrm{CI}}_{i}}Llh_{0}B^{2}}{2\mu_{0}};\;\left(i\;=1,2,3\right),$$

where $\Phi_{\mathrm{CI}}\;\left(i=1,2,3\right)$ is the volume fraction of the magnetic dipoles, identically equal to the volume fraction of the carbonyl iron microparticles from Table 3; *L*, *l,* and $h_{0}$ are the length, width, and thickness of the membranes $M_{i}\;\left(i=1,2,3\right),\;\mathrm{respectively},$ at the moment of applying the magnetic field (*t* = 0 s); and $\mu_{0}$ is the magnetic permeability of the vacuum.

If, at the time *t* = 0, the thickness of the membranes $M_{i}\;\left(i=1,2,3\right)$ is $h_{0}$, then at a time *t* ≫ 0 after application of the magnetic field, the thickness of the membranes under the action of $F_{m_{i}}\;\left(i=1,2,3\right)$ becomes $h_{i}<h_{0}\;\left(i=1,2,3\right)$.

It is known [25,26] that, in magnetorheological elastomers located in a magnetic field, there are interactions between the magnetic dipoles and between the magnetic dipoles and the respective silicone rubber that keeps them coupled. The result of the intensity of these interactions represents the resistance force opposed by the magnetorheological elastomer to the magnetic force.

Adopting this model for the membranes $M_{i}\;\left(i=1,2,3\right)$, which are considered continuous and deformable bodies, the force opposing the magnetic force, $F_{m_{i}}$, is the resistance force $F_{r_{i}}$. It acts in the same direction but opposite to the magnetic force.

The value of the force $F_{r_{i}}$ is calculated using the following equation [26,27]:

(5) $$F_{r_{i}}=-k_{i}\left(h_{i}-h_{0\;}\right);\;\left(i=1,2,3\right),$$

where $k_{i}$ is the magneto-deformation coupling constant of the membranes $M_{i}\;\left(i=1,2,3\right)$, $h_{i\;}\;\left(i=1,2,3\right)$ is the thickness of Ms at the moment *t* ≫ 0 after the application of *B*, and $h_{0\;}$ is the thickness of the same Ms but at the moment *t* = 0 of magnetic field application.

At the moment *t* = Δ*t* between the forces $F_{m_{i}}$ and $F_{r_{i}}$ (i=1,2,3), a balance is achieved which is mathematically expressed by the equality $F_{m_{i}}=-F_{r_{i}}$ (with *i* = 1,2, and 3). In this equality, we insert Equations (4) and (5) to obtain:

(6) $$h_{i}=h_{0\;}\left(1-\frac{9\Phi_{{\mathrm{CI}}_{i}}LlB^{2}}{2\mu_{0}k_{i}}\right);\;\left(i=1,2,3\right),$$

where the notation is as outlined above.

It can be seen from Equation (6) that when the magnetic field is applied, the values of the thicknesses $h_{i}\;\left(i=1,2,3\right)$ decrease significantly with the increase in the magnetic field strength. Additionally, $h_{i}\;\left(i=1,2,3\right)$ is dependent on the volume fraction values of CI microparticles contained in the honey impregnated in cotton microfibers, $\Phi_{\mathrm{CI}}\;\left(i=1,2,3\right)$.

Considering the devices ${\mathrm{ED}}_{i}\;\left(i=1,2,3\right)$ located in the magnetic field as linear resistors, we can calculate their electrical resistance using the following equation [26,27,28]:

(7) $$R_{i}=\frac{U}{I_{i}}=\frac{h_{i}}{\sigma_{0}{}_{i}\cdot L\cdot l};\;\left(i=1,2,3\right),$$

where U is the electric voltage, $I_{i}\;\left(i=1,2,3\right)$ is the electric current intensity through the resistors ${\mathrm{ED}}_{i}\;\left(i=1,2,3\right)$ at the moment *t* ≫ 0 after the application of *B*, $h_{i\;}\;\left(i=1,2,3\right)$ is the thickness of Ms at moment *t* ≫ 0 after the application of *B*, $h_{0\;}$ is the thickness of the same Ms but at the moment (*t* = 0) of magnetic field application, and $\sigma_{0}{}_{i}\;\left(i=1,2,3\right)$ is the conductivity of Ms, from Table 3.

From Equations (6) and (7), we can obtain the formula for calculating the electrical resistance of devices ${\mathrm{ED}}_{i}\;\left(i=1,2,3\right)$ subjected to a magnetic field as

(8) $$R_{i}=R_{0_{i}\;}\left(1-\frac{9\Phi_{{\mathrm{CI}}_{i}}LlB^{2}}{2\mu_{0}k_{i}}\right);\;\left(I\;=1,2,3\right),$$

where $R_{0_{i}\;}\left(i=1,2,3\right)$ is the electrical resistance of devices ${\mathrm{ED}}_{i}\;\left(i=1,2,3\right)$ in the absence of the magnetic field given by

(9) $$R_{0_{i}}=\frac{U}{I_{0_{i}}}=\frac{h_{0}}{\sigma_{0}{}_{i}\cdot L\cdot l};\;\left(i=1,2,3\right)$$

where the notation is as outlined above.

If a continuous and constant voltage *U* is applied to the terminals of $R_{i}\;\left(i=1,2,3\right)$, then an electric current passes through the resistor ${\mathrm{ED}}_{i}\;\left(i=1,2,3\right)$ with the intensity given by

(10) $$I_{i}=I_{0_{i}\;}{\left(1-\frac{9\Phi_{{\mathrm{CI}}_{i}}LlB^{2}}{2\mu_{0}k_{i}}\right)}^{-1};\;\left(i=1,2,3\right),$$

where the notation is as outlined above.

We note that Equation (10) qualitatively describes the results obtained in Figure 8a. This statement must be understood in the sense that, in the absence of the magnetic field, the values $I_{0_{i}\;}\left(i=1,2,3\right)$ for the constant values of the electric voltage are determined by the composition of the membranes $M_{i}\;\left(i=1,2,3\right)$ (see Table 3). On the other hand, from (10), it can be observed that for the case of devices ${\mathrm{ED}}_{i}\;\left(i=1,2,3\right)$ located in a magnetic field, the values of $I_{i}\;\left(i=1,2,3\right)$ are strongly influenced by the B values of the magnetic field density, as we experimentally obtained in Figure 8a.

The magneto-deformation coupling constant of the membranes $M_{i}\;\left(i=1,2,3\right)$, $k_{i}\;\left(i=1,2,3\right)$, extracted from Relation (10), will be

(11) $$k_{i}=\frac{9\Phi_{{\mathrm{CI}}_{i}}LlB^{2}}{2\mu_{0}\left(1-\frac{I_{0_{i}}}{I_{i}}\right)};\;\left(i=1,2,3\right),$$

where the notation is as outlined above.

When L=30 mm, $l=25\;\mathrm{mm},\;\mu_{0}=4\pi \cdot 10^{-7}H/m,$ and the values of $\Phi_{{\mathrm{CI}}_{i}}\;\left(i=1,2,3\right)$ from Table 3 are inserted into Equation (11), we have

(12) $$10^{3}\cdot k(N/m)=\{\begin{array}{c} \frac{0.2296\cdot B^{2}\left(\mathrm{mT}\right)}{1-\frac{I_{0_{1}}}{I_{1}}\;};\;\mathrm{for}\;M_{1}\\ \frac{0.2053\cdot B^{2}\left(\mathrm{mT}\right)}{1-\frac{I_{0_{2}}}{I_{2}}\;};\;\mathrm{for}\;M_{2}\\ \frac{0.1886\cdot B^{2}\left(\mathrm{mT}\right)}{1-\frac{I_{0_{3}}}{I_{3}}\;};\;\mathrm{for}\;M_{3} \end{array},$$

where the notation is as outlined above.

**Figure 9:**
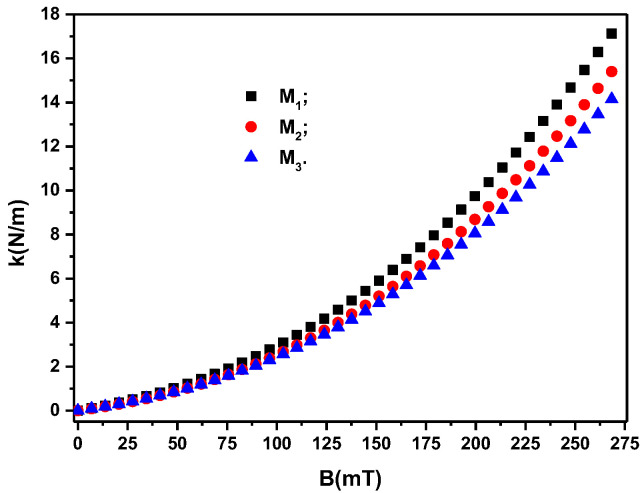
The magneto-deformation coupling constant, *k*, of the membranes $M_{i}\;\left(i=1,2,3\right)$ depending on the values of the magnetic flux density, *B*.

If, in the group of Relation (12), we introduce the $I=I{\left(B\right)}_{i}\;\left(i=1,2,3\right)$ from Figure 8a, we obtain the $k=k{\left(B\right)}_{i}\;\left(i=1,2,3\right)$, as shown in Figure 9. 

It can be seen from Figure 9 that the quantities $k_{i}\;\left(i=1,2,3\right)$ appear when a magnetic field is applied. Their value depends on the surface area of the membranes and on the volume fractions of the magnetic dipoles, $\Phi_{\mathrm{CI}}\;\left(i=1,2,3\right)$. On the other hand, for the same membrane geometry and the same values of $\Phi_{\mathrm{CI}}\;\left(i=1,2,3\right)$, the values of $k_{i}\;\left(i=1,2,3\right)$ depend significantly on the density of the magnetic flux, *B*. In addition, from Figure 9, it can be seen that by adding silver microparticles, such as in the case of membranes $M_{2}$ and $M_{3}$, and reducing the volume fraction of microparticles CI (see Table 3), the value of *k* decreases relative to that of membrane $M_{1}$ for the same values of *B*. This result suggests that we can consider the columns of dipoles as chains arranged in an orderly manner in the volume of the membranes $M_{i}\;\left(i=1,2,3\right)$. The stiffness of the chains is dependent on the volume fraction of the magnetic dipoles and the values of the magnetic flux density *B*. This result suggests that we can consider the columns with chain dipoles as being arranged in an orderly manner in the volume of the membranes $M_{i}\;\left(i=1,2,3\right)$, as reported in [28] for magnetorheological elastomers. 

The decrease in the value of the volume fraction of the magnetic dipoles (see Table 3) denotes the reduction in the number of chains and, consequently, the reduction in the value of *k* for the membranes $M_{2}$ and $M_{3}$ compared with that of membrane $M_{1}$ for the same value of *B*, as can be seen from Figure 9. If we introduce $k=k{\left(B\right)}_{i}\;\left(i=1,2,3\right)$ from Figure 9 into Equation (10), we obtain the polynomial fits in Figure 8a, which are a good approximation of the experimental data. 

Further, we can use Equation (3), adapted for the case of membranes $M_{i}\;\left(i=1,2,3\right)$ placed in a magnetic field, in which we introduce the $I=I{\left(B\right)}_{i}\;\left(i=1,2,3\right)$ from Figure 8a to obtain $\sigma =\sigma {\left(B\right)}_{i}\;\left(i=1,2,3\right)$ represented in Figure 8b. It can be seen from Figure 8b that the plots of function $\sigma =\sigma {\left(B\right)}_{i}$ have the same shape and dependence on the volume fractions of conductive microparticles and the magnetic flux density values as the corresponding plots of $I=I{\left(B\right)}_{i}\;\left(i=1,2,3\right)$ in Figure 8a. 

From Table 4, it can be seen that the membranes $M_{i}\;\left(i=1,2,3\right)$ are characterized by an intrinsic or own electrical conductivity due to their composition (see Table 3) and an apparent electrical conductivity (Figure 8a,b) as an effect of the induction of magneto-deformations in Ms placed in the magnetic field, as given by Equations (6) and (9). Indeed, according to Equation (6), in the membranes placed in the magnetic field, the forces $F_{m_{i}}\;\left(i=1,2,3\right)$ induce deformations in the direction of the Oz coordinate axis with the following components:

(13) $$\varepsilon_{zz_{i}}=\frac{h_{i}}{h_{0}}-1;\;\left(i=1,2,3\right),$$

where $h_{i}\;\left(i=1,2,3\right)$ and $h_{0}$ are the thicknesses of the membranes $M_{i}\;\left(i=1,2,3\right)$ in the presence and absence of the magnetic field, respectively.

If, in Equation (13), we replace $h_{i}$ and $h_{0}$ from the definitions (7) and (9) of the electrical resistances in the presence and absence of the magnetic field, we can obtain the components of the deformations as

(14) $$\varepsilon_{zz_{i}}=\frac{I_{0_{i}}}{I_{i}}-1;\;\left(i=1,2,3\right).$$

**Figure 10:**
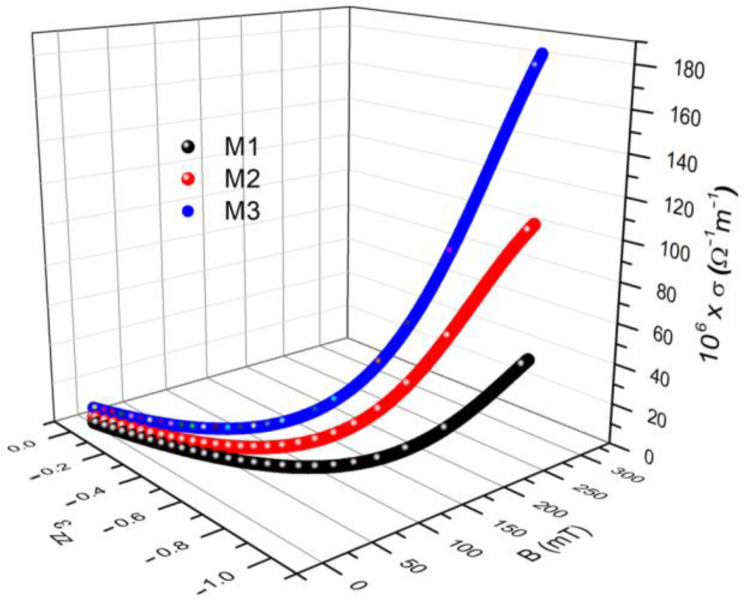
The electrical conductivity *σ* and the components $\varepsilon_{zz}$ of the deformations induced in the membranes $M_{i}\;\left(i=1,2,3\right)$ depending on the values of the magnetic flux density.

We insert the $I=I{\left(B\right)}_{i}\;\left(i=1,2,3\right)$ from Figure 8a into Equation (14) to obtain $\varepsilon_{zz_{i}}=\varepsilon_{zz_{i}}\left(B\right)\;\left(i=1,2,3\right)$. For each value of the magnetic flux density, *B*, there are corresponding components of the deformations and values of the electrical conductivity in Figure 8b. Similar results were reported for the case of magnetorheological elastomers [29,30], and for hydrogels [31]. The deformations $\varepsilon_{zz_{i}}$ correspond to the electrical conductivity values from Figure 8b, at the same value as the magnetic field density, *B*. The *σ* values are due to the electronic conduction achieved through the tunnel effect [27,32]. As reported in [27,32], by increasing the values of the magnetic field density, *B*, the height of the electrical potential well decreases so that the electrons from lower energy levels can pass over. The effect is the increase of electrical conductivity with the increase of *B*. Through the 3D representation of the coordinate points (*B*, *σ*, $\varepsilon_{zz}$), we obtain $\sigma =\sigma {\left(B,\;\varepsilon_{zz}\right)}_{i}\;\left(i=1,2,3\right)$, as shown in Figure 10. 

It can be seen from Figure 10 that for each value of the magnetic flux density, *B*, deformations with the components $\varepsilon_{zz_{i}}\;\left(i=1,2,3\right)$ and apparent electrical conductivities of $\sigma_{i}\;\left(i=1,2,3\right)$ are induced in the membranes $M_{i}\;\left(i=1,2,3\right)$. From the same figure, we can see that the coordinate points (*B*, *σ*, $\varepsilon_{zz}$) depend on the composition of the membranes from Table 3 and on the values of the magnetic flux density *B*. To highlight the effect of the magnetic field on electrical conductivity, we extracted the numerical values from the 3D graph shown in Figure 10 for two extreme values of the magnetic field, as shown in Table 5. 

It was observed that by increasing the value of the magnetic flux density, as an effect of the magneto-deformation of the membranes (see Equation (6)), the components of the deformations as well as the values of the apparent electrical conductivity increase in absolute value.

## 4. Conclusions

In this paper, we report on the manufacture of four membranes, $M_{i}\;\left(i=0,1,2,3\right)$ using cotton microfibers impregnated with honey alone or honey with different concentrations of carbonyl iron microparticles and silver microparticles (Figure 2). SEM microscopy revealed that the metal microparticles with bee honey and cotton microfibers form a common body (Figure 4a,b). Using epoxy resin plates plated with copper foil, electrical devices, ${\mathrm{ED}}_{i}\;\left(i=0,1,2,3\right)$, were made from membranes. Using a specially designed experimental set-up (Figure 7), at the terminals of the EDs, the voltage $U=2{\;V}_{\mathrm{dc}}$ was fixed, and in the absence of the magnetic field the values, I, of the electric current intensity were measured. The experimental data regarding the intensity of the electric current through the EDs suggested that these electrical devices can be considered linear electrical resistors through which the intensity of the continuous electric current is controlled by the magnetic field for constant values of electric voltage (Figure 8a). The electrical conductivity of the membranes $M_{i}\;\left(i=1,2,3\right)$ comprised two components: one of intrinsic conductivity due to the composition (Table 4), and the second is the apparent electrical conductivity, which appears as an effect of the magneto-deformation of the membranes under the action of the magnetic field (Figure 10). In the absence of the magnetic field, the electrical conductivity increased from ~3.42 times to ~12.55 times due to the increase in mass of the metal microparticles inserted in the membranes. The contribution brought by silver microparticles to the increase in electrical conductivity was from ~2.25 times to ~3.67 times, when doubling the silver microparticle mass. When applying a magnetic field, the electrical conductivity of the membranes with microparticles of carbonyl iron and silver, $M_{i}\;\left(i=1,2,3\right)$, increases with increasing magnetic flux density *B* (Table 5). For the membrane with the mass ratio of microparticles 1:1 in a magnetic field, the increase in electrical conductivity was up to 180 times higher than that of the membrane based on cotton fabric with honey alone.

It was shown that the electrical conductivity values of the membranes can be roughly adjusted by the composition and fine-tuned by the magnetic field (Figure 8a,b). Thus, the membranes reported in this study are new low-cost, biodegradable, and efficient magnetizable membranes with electrical properties that can be preset in advance by choosing the right concentration of components and a certain value of density for the applied magnetic field. 

## Figures and Tables

**Table 1:** Liquid magnetic suspensions.

$S_{i}$	$m_{\mathrm{HB}}\;\left(g\right)$	$m_{\mathrm{CI}}\;\left(g\right)$	$m_{\mathrm{SmP}}\;\left(g\right)$	$\varphi_{\mathrm{HB}}\;\left(w\%\right)$	$\varphi_{\mathrm{CI}}\;\left(w\%\right)$	$\varphi_{\mathrm{SmP}}\;\left(w\%\right)$
$S_{0}$	1.20	0.00	0.00	100.00	0.00	0.00
$S_{1}$	1.20	0.40	0.00	75.00	25.00	0.00
$S_{2}$	1.20	0.40	0.20	66.67	22.22	11.11
$S_{3}$	1.20	0.40	0.40	60.00	20.00	20.00

**Table 2:** Masses of components in the membranes $M_{i}\;\left(i=0,1,2,3\right)$ .

$M_{i}$	$m_{\mathrm{GB}}\;\left(g\right)$	$m_{\mathrm{HB}}\;\left(g\right)$	$m_{\mathrm{CI}}\;\left(g\right)$	$m_{\mathrm{SmP}}\;\left(g\right)$
$M_{0}$	0.200	0.776	0.000	0.000
$M_{1}$	0.200	0.776	0.258	0.000
$M_{2}$	0.200	0.776	0.258	0.129
$M_{3}$	0.200	0.776	0.258	0.258

**Table 3:** Volumes and volume fractions for the components of membranes $M_{i}\;\left(i=0,1,2,3\right)$.

$M_{i}$	$V_{f}\;\left({\mathrm{cm}}^{3}\right)$	$V_{\mathrm{HB}}\;\left({\mathrm{cm}}^{3}\right)$	$V_{\mathrm{CI}}\;\left({\mathrm{cm}}^{3}\right)$	$V_{\mathrm{SmP}}\;\left({\mathrm{cm}}^{3}\right)$	$\Phi_{f}\;\left(\mathrm{vol}.\%\right)$	$\Phi_{\mathrm{HB}}\;\left(\mathrm{vol}.\%\right)$	$\Phi_{\mathrm{CI}}\;\left(\mathrm{vol}.\%\right)$	$\Phi_{\mathrm{SmP}}\;\left(\mathrm{vol}.\%\right)$
$M_{0}$	0.2720	0.5187	0.0000	0.0000	34.39989	65.60011	0.00000	0.000000
$M_{1}$	0.2720	0.5187	0.0735	0.0000	31.47430	60.02080	8.50490	0.000000
$M_{2}$	0.2720	0.5187	0.0735	0.0966	28.30970	53.98630	7.64499	10.05410
$M_{3}$	0.2720	0.5187	0.0735	0.1932	25.72350	49.05420	6.95100	18.27130

**Table 4:** The electric current intensities, *I*, through Eds, and the conductivities $\sigma_{0}{}_{i}\;\left(i=0,1,2,3\right)$ of Ms at B = 0 mT.

${\mathrm{ED}}_{i}$	$I\left(\mu A_{\mathrm{dc}}\right)$	$M_{i}$	$10^{6}\cdot \sigma_{0}{}_{i}\left(\Omega^{-1}\cdot m^{-1}\right)$
${\mathrm{ED}}_{0}$	2.40	$M_{0}$	0.64
${\mathrm{ED}}_{1}$	8.20	$M_{1}$	2.19
${\mathrm{ED}}_{2}$	18.50	$M_{2}$	4.93
${\mathrm{ED}}_{3}$	30.10	$M_{3}$	8.03

**Table 5:** The components of deformations, $\varepsilon_{zz_{i}}\;\left(i=1,2,3\right)$, and the apparent electrical conductivities, $\sigma_{i}\;\left(i=1,2,3\right)$, of the membranes $M_{i}\;\left(i=1,2,3\right)$ for two values of magnetic field.

*B* (mT)	$M_{i}$	$\varepsilon_{zz}$	$10^{6}\cdot \sigma \;\left(\Omega^{-1}\cdot m^{-1}\right)$
25	$M_{1}$	−0.32236	2.11025
	$M_{2}$	−0.35263	6.50703
	$M_{3}$	−0.31506	13.12206
275	$M_{1}$	−0.96701	43.34193
	$M_{2}$	−0.96109	108.25721
	$M_{3}$	−0.95140	184.99065

## Data Availability

Experimental data are available by reasonable requests from the authors.

## Abbreviations

The following abbreviations are used in this manuscript:

Ms	Membranes
GB	Sterile compresse
CmF	Cotton microfibers
HB	Bee honey
CI	Carbonyl iron
SmP	Silver microparticles
ED	Electrical device
